# LncRNA KCNQ1OT1 promotes cell proliferation, migration and invasion via regulating miR-129-5p/JAG1 axis in non-small cell lung cancer

**DOI:** 10.1186/s12935-020-01225-8

**Published:** 2020-05-01

**Authors:** Yan Wang, Lei Zhang, Jiasheng Yang, Ruilin Sun

**Affiliations:** 1 Department of Pulmonary and Critical Care Medicine, The Guangdong Second Provincial General Hospital, No. 466 Xingang Middle Rd, Haizhu District, 510000 Guangzhou, China; 2 Department of Transplant Centre, The Second Affiliated Hospital of Guangzhou Medical University, 510000 Guangzhou, China

**Keywords:** KCNQ1OT1, miR-129-5p, JAG1, Non-small cell lung cancer

## Abstract

**Background:**

Non-small cell lung cancer (NSCLC) is the most deadly cancer worldwide. LncRNA KCNQ1OT1 has been reported to be involved in the progression of various tumors, including NSCLC. However, the precise mechanism of KCNQ1OT1 in NSCLC requires further investigation.

**Methods:**

The expression levels of KCNQ1OT1, miR-129-5p and JAG1 were detected by qRT-PCR or western blot. Kaplan–Meier survival analysis was used to assess the correlation between KCNQ1OT1 expression and the overall survival of NSCLC patients. CCK-8 assay was used to measure cell viability. Cell migration and invasion were detected by transwell assay. The targets of KCNQ1OT1 and miR-129-5p were predicted by bioinformatics, which was confirmed by dual-luciferase reporter assay or pull-down assay.

**Results:**

KCNQ1OT1 expression was significantly enhanced, while miR-129-5p expression was dramatically reduced in NSCLC tissues and cells. Higher KCNQ1OT1 shortened overall survival and was positively associated with tumor stage and lymph node metastasis. KCNQ1OT1 knockdown inhibited proliferation, migration and invasion of NSCLC cells. Inhibition of miR-129-5p attenuated the inhibition of NSCLC cell viability, migration and invasion induced by KCNQ1OT1 knockdown. In addition, JAG1 was confirmed as a target of miR-129-5p. Knockdown of JAG1 reversed the effects of miR-129-5p knockdown on NSCLC progression. KCNQ1OT1 regulated JAG1 expression by sponging miR-129-5p in NSCLC cells.

**Conclusion:**

KCNQ1OT1 induced proliferation, migration and invasion of NSCLC cells by sponging miR-129-5p and regulating JAG1 expression, indicating that KCNQ1OT1 was a therapeutic target for NSCLC.

## Background

Lung cancer is the most deadly cancer in the world with high morbidity and mortality [1]. Lung cancer is divided into two subtypes of non-small cell lung cancer (NSCLC) and small cell lung cancer (SCLC). Non-small cell lung cancer (NSCLC) is the most common subtype, accounting for more than 80% of all lung cancer [2]. Moreover, the prognosis of NSCLC patients is still poor, with a 5-year survival rate lower than 18% [3]. Although the research of NSCLC has been made progress in the past, the molecular mechanism of NSCLC still needs to be explored.

Long noncoding RNAs (lncRNAs) are non-protein coding transcripts longer than 200 nucleotides in length [4]. Increasing evidence has shown that lncRNAs exerted significant regulatory effects on various biological processes and gene expression through multiple mechanisms [5]. KCNQ1 overlapping transcript 1 (KCNQ1OT1) was involved in the regulation of numerous genes within the kcnq1 domain [6]. LncRNA KCNQ1OT1 has been reported to play roles in the pathogenesis of various cancer, such as colorectal cancer [7], breast cancer [8] and tongue cancer [9]. In previous studies, KCNQ1OT1 facilitated progression of cholangiocarcinoma (CCA) by sponging miR-140-5p and regulating SOX4 expression [10]. However, the mechanism of KCNQ1OT1 in NSCLC still needs further research.

MicroRNAs (miRNAs) are non-protein-encoded short noncoding RNAs composed of 18-25 nucleotides. The roles of miRNAs in epigenetic regulation had progressed, in which miRNAs regulated the protein levels of target mRNAs [11]. A previous study revealed that miR-129-5p was down-regulated, and inhibited cell proliferation and EMT by negatively regulating HMGB1 in gastric cancer [12]. Moreover, the role of miR-129-5p in NSCLC have also been studied. LncRNA NNT-AS1 was a carcinogen in non-small cell lung cancer (NSCLC), and acted as a competing endogenous RNA (ceRNA) by targeting miR-129-5p in lung cancer [13]. However, the relationship between KCNQ1OT1 and miR-129-5p in the progression of NSCLC has not been elucidated.

Jagged1 (JAG1) has been reported to play a role in multiple types of cancer [14]. Previous studies have shown that JAG1 regulated tumor progression, including NSCLC. Tang et al. found that miR-377-3p suppressed cell proliferation and invasion via targeting JAG1 in ovarian cancer [15]. Wang et al. indicated that miR-26b was a tumor inhibitor through binding to JAG1 in cervical cancer [16].

In this study, we measured the expression levels of KCNQ1OT1, miR-129-5p and JAG1 in NSCLC tissues and cells. In addition, we explored the potential interactions between KCNQ1OT1 and miR-129-5p or miR-129-5p and JAG1. In conclusion, this study may provide novel therapeutic targets for NSCLC treatment.

## Materials and methods

### Tissue samples

All NSCLC tissues and the corresponding adjacent normal tissues were obtained from patients who underwent surgical resection at the Guangdong Second Provincial General Hospital. All patients did not undergo treatments prior to surgery. All patients were categorized according to the Eighth Edition of the American Joint Committee on Cancer TNM Staging System for lung cancer. This research was approved by the Ethics Committee of the Guangdong Second Provincial General Hospital, and written informed consent was collected from all participants. All tissue samples were immediately frozen in liquid nitrogen and then stored at − 80 °C until RNA extraction. The clinical characteristics of patients are summarized in Table 1.

**Table 1:** Correlation between KCNQ1OT1 expression and clinical characters in 60 patients with NSCLC

Characteristic	Case number	KCNQ1OT1	expression	*P* value
		High	Low	
Age				0.7892
≥ 60	38	18	20	
< 60	22	12	10	
Gender				0.6042
Man	33	18	15	
Woman	27	12	15	
Smoking				0.4118
Yes	40	22	18	
No	20	8	12	
Histology				0.2949
Adenocarcinoma	35	20	15	
Squamous carcinoma	25	10	15	
Stage				0.0379
I + II	31	11	20	
III + IV	29	19	10	
Lymph node metastasis				0.0127
Positive	40	25	15	
Negative	20	5	15	

### Cell culture

Human lung epithelial cell line (BEAS-2B) and the NSCLC cell lines (A549, H1299, H460, H446 and H1975) were purchased from ATCC (American Type Culture Collection, Manassas, VA, USA) and were grown in Dulbecco’s Modified Eagle Medium (DMEM; Thermo Fisher Scientific, Waltham, MA, USA) supplemented with 10% fetal bovine serum (FBS; Gibco, Carlsbad, CA, USA) at 37 °C containing 5% CO_2_.

### Cell transfection

Small interfering RNA (siRNA) against KCNQ1OT1 (si-KCNQ1OT1, 5′-GGUAGAAUAGUUCUGUCUU-3′; si-KCNQ1OT1#2, 5′-GCCAAUAGCAACUGACUAA-3′; si-KCNQ1OT1#3, 5′-GCCACAUCUAACACCUAUA-3′) and the negative control siRNA (si-NC), KCNQ1OT1 overexpression plasmid (pcDNA-KCNQ1OT1) and the control (pcDNA-NC) were purchased from RiboBio (Guangzhou, China). The miR-129-5p mimic and the mimic negative control (NC mimic), miR-129-5p inhibitor and the negative control (NC inhibitor), siRNA against JAG1 (si-JAG1, 5′-GGCCAAGCCUUGUGUAAAU-3′) and the corresponding negative control (si-NC) were synthesized by Genelily BioTech (Shanghai, China). Cells were transfected by Lipofectamine 2000 (Invitrogen, Carlsbad, CA, USA) following the manufacturer’s requirements.

### Quantitative real-time PCR

Total RNA was isolated from tissues and cells using TRIzol reagent (Invitrogen) by the protocols of the manufacturer. The first strand cDNA was synthesized using the High-Capacity cDNA Reverse Transcription Kits (Thermo Fisher Scientific). The expression levels were detected using SYBR Green Mixture (Takara, Dalian, China). GAPDH or U6 was used as the endogenous control. Primers as follows: KCNQ1OT1 (forward, 5′-AGGGTGACAGTGTTTCATAGGCT-3′; reverse, 5′-GAGGCACATTCATTCGTTGGT-3′), miR-129-5p (forward, 5′-ACCCAGTGCGATTTGTCA-3′; reverse, 5′-ACTGTACTGGAAGATGGACC-3′), JAG1 (forward, 5′-GTCCATGCAGAACGTGAACG-3′; reverse, 5′-GCGGGACTGATACTCCTTGA-3′), GAPDH (forward, 5′-TCGCCAGCCGAGCCACATC-3′; reverse, 5′-CGTTCTCAGCCTTGACGGTGC-3′), U6 (forward, 5′-CGATACAGAGAAGATTAGCATGGC-3′; reverse, 5′-AACGCTTCACGAATTTGCGT-3′).

### CCK-8 assay

The viability of cells was detected with Cell Counting Kit-8 (CCK-8; Dojindo, Kumamoto, Japan). First, 100 µl of cell suspension (1.0 × 10^5^ cells per well) were seeded into 96-well plates and cultured at 37 °C. Then, 10 µl CCK-8 solution was added to each well after incubation for 48 h. After 4 h, the absorbance was read at 450 nm using Microplate Reader (Bio-Rad, Hercules, CA, USA).

### Transwell assay

Cell migration and invasion ability were detected using transwell assay. For cell migration, the transfected cells were plated (1.0 × 10^5^ cells per well) in the upper chamber of a 24-well transwell containing 8 μm polycarbonate membrane (Millipore, Billerica, MA, USA). The serum-free DMEM was added into the upper chamber, and DMEM containing 10% FBS as a chemoattractant was added to the lower chamber. Then, cells were cultured for 48 h at 37 °C, and the cells migrated to the lower surface were fixed with methanol and stained with 0.1% crystal violet. At last, the cells were counted with microscopy. For cell invasion, transwell chambers were coated with Matrigel (Millipore), and other experimental procedures were performed as previously described.

### Dual-luciferase reporter assay

The putative binding sites of KCNQ1OT1 and miR-129-5p or miR-129-5p and JAG1 were predicted by LncBase Predicted v.2 or StarBase v2.0. The fragments of KCNQ1OT1 containing wild-type (wt) or mutant (mut) binding sites of miR-129-5p were inserted into pGL3 luciferase reporter plasmids (Promega, Madison, WI, USA), and were co-transfected with miR-129-5p mimic or NC mimic into A549 and H460 cells using Lipofectamine 2000 (Invitrogen). In addition, the sequences of JAG1 3′UTR containing wild-type (wt) or mutant (mut) binding sites of miR-129-5p was cloned into pGL3 vectors (Promega). A549 and H460 cells were co-transfected with miR-129-5p mimic or NC mimic and corresponding luciferase reporter vector using Lipofectamine 2000 (Invitrogen). After the transfection for 48 h, Dual-Luciferase Reporter Assay System (Promega) was used for luciferase activity analysis according to the manufacturer’s instructions.

### RNA pull-down assay

RNA pull-down assay was performed as previously described [17]. Biotin-labeled wild-type KCNQ1OT1 (Bio-KCNQ1OT1), mutant KCNQ1OT1 (Bio-KCNQ1OT1-MUT) and the control (Bio-NC) were purchased from RiboBio. Briefly, the biotinylated RNA was incubated with total RNA extracted from A549 and H460 cell lysates overnight at 4 °C. Then, the biotin-coupled RNA complexes were pulled down using M-280 Streptavidin Dynabeads (Invitrogen) at room temperature for 2 h. After RNA isolation, the abundance of miR-129-5p was detected by qRT-PCR.

### Western blot assay

After transfection, A549 and H460 cells were harvested and lysed in RIPA lysis buffer (Thermo Fisher Scientific). Total protein was quantified using the BCA Protein Assay Kit (Pierce, Appleton, WI, USA) with the protocols of the manufacturer. Then, the proteins were separated by SDS-PAGE and transferred to polyvinylidene fluoride (PVDF) membranes (Millipore). Furthermore, the membranes were blocked by 5% non-fat milk (Nestlé, Shuangcheng, China) for 2 h at 37 °C, and then incubated with primary antibodies against JAG1 (1:2000; Abcam, Cambridge, UK) or GAPDH (1:2000; Abcam) overnight at 4 °C. Subsequently, the membranes were washed three times with TBST and interacted with horseradish peroxidase (HRP)-conjugated secondary antibody (1:4000; Abcam) for 2 h at room temperature. Finally, the protein bands were visualized via enhanced chemiluminescence system (Thermo Fisher Scientific) and quantitated by ImageJ software (National Institutes of Health, Bethesda, MD, USA). GAPDH was regarded as an endogenous reference.

### Statistical analysis

All data were displayed as the mean ± standard deviation (SD) from three independent experiments. Differences between the two groups were analyzed by Student’s *t* test, and multiple groups were analyzed by one-way analysis of variance (one-way ANOVA). Statistical analysis was performed using Graphpad Prism 7.0 software (GraphPad, San Diego, CA, USA). At *P*-value < 0.05, the difference was considered to be statistically significant.

## Results

### KCNQ1OT1 was upregulated in NSCLC tissues and cells and correlated with poor prognosis

**Fig. 1:**
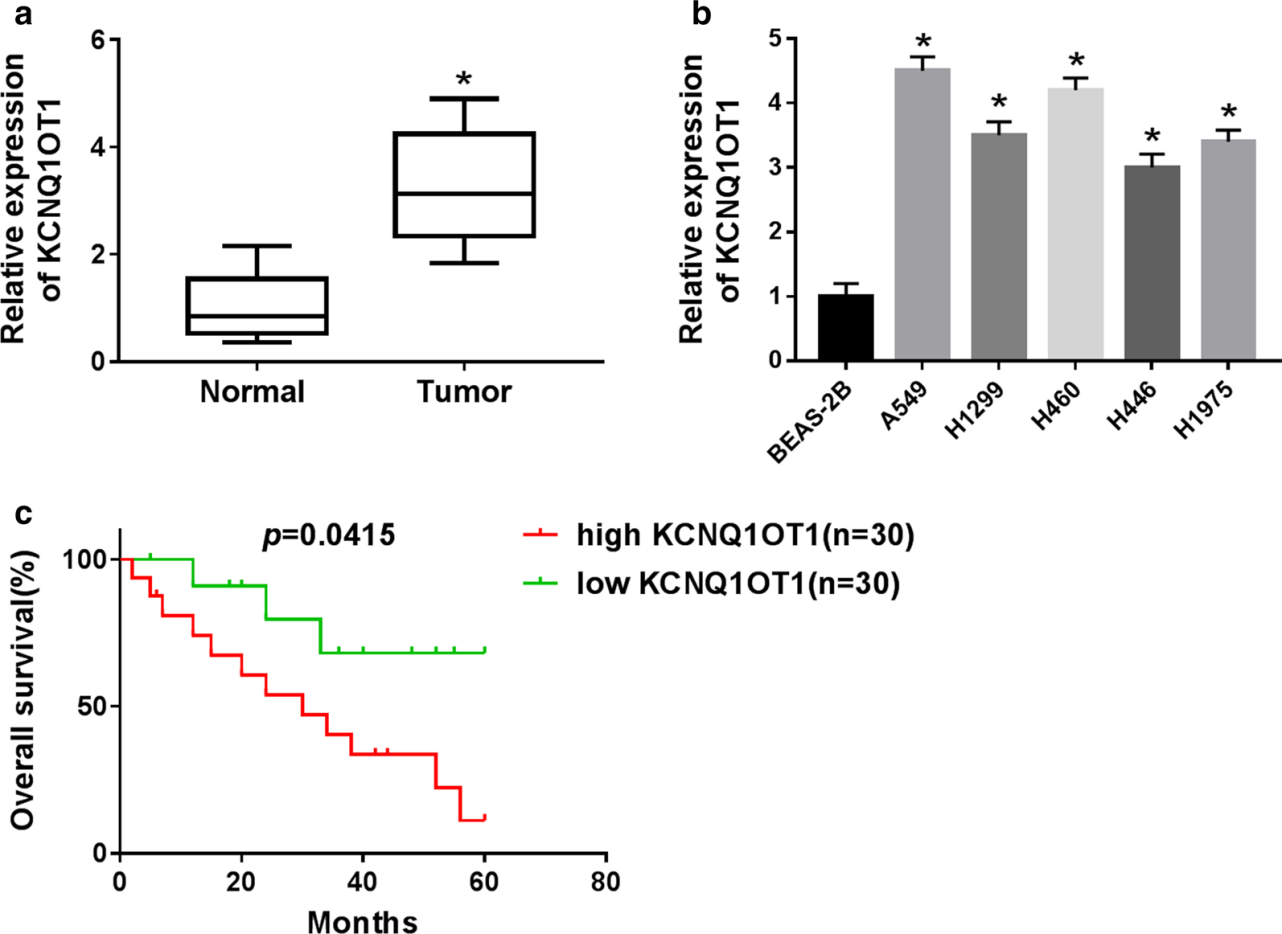
KCNQ1OT1 was upregulated in NSCLC tissues and cells and correlated with poor prognosis. **a** qRT-PCR was used to detect KCNQ1OT1 expression in NSCLC tissues and adjacent normal tissues. **b** The KCNQ1OT1 expression was detected in normal lung epithelial cell line (BEAS-2B) and the NSCLC cell lines (A549, H1299, H460, H446 and H1975) by qRT-PCR. **c** Kaplan–Meier survival analysis was performed to investigate the correlation between KCNQ1OT1 expression and overall survival rate of NSCLC patients. *P < 0.05

In order to verify the differential expression of KCNQ1OT1 in NSCLC tissues and cells, qRT-PCR was performed to detect expression levels. The results revealed that KCNQ1OT1 expression was significantly upregulated in NSCLC tissues compared to adjacent non-tumor tissues (Fig. 1a). Similarly, the expression of KCNQ1OT1 was dramatically higher in NSCLC cells (A549, H1299, H460, H446 and H1975) than that in BEAS-2B cells (Fig. 1b). Furthermore, Kaplan–Meier survival analysis showed that higher KCNQ1OT1 expression resulted in poor overall survival compared with lower KCNQ1OT1 expression (Fig. 1c). Finally, we also investigated the correlation between KCNQ1OT1 expression levels and clinical pathological features. The data indicated that KCNQ1OT1 expression was not associated with patient age, gender, smoking and histology, but was correlated with tumor stage and lymph node metastasis (Table 1). All these data suggested that KCNQ1OT1 expression was related to NSCLC prognosis and might play crucial roles in NSCLC development and progression.

### KCNQ1OT1 knockdown inhibited proliferation, migration and invasion of NSCLC cells

**Fig. 2:**
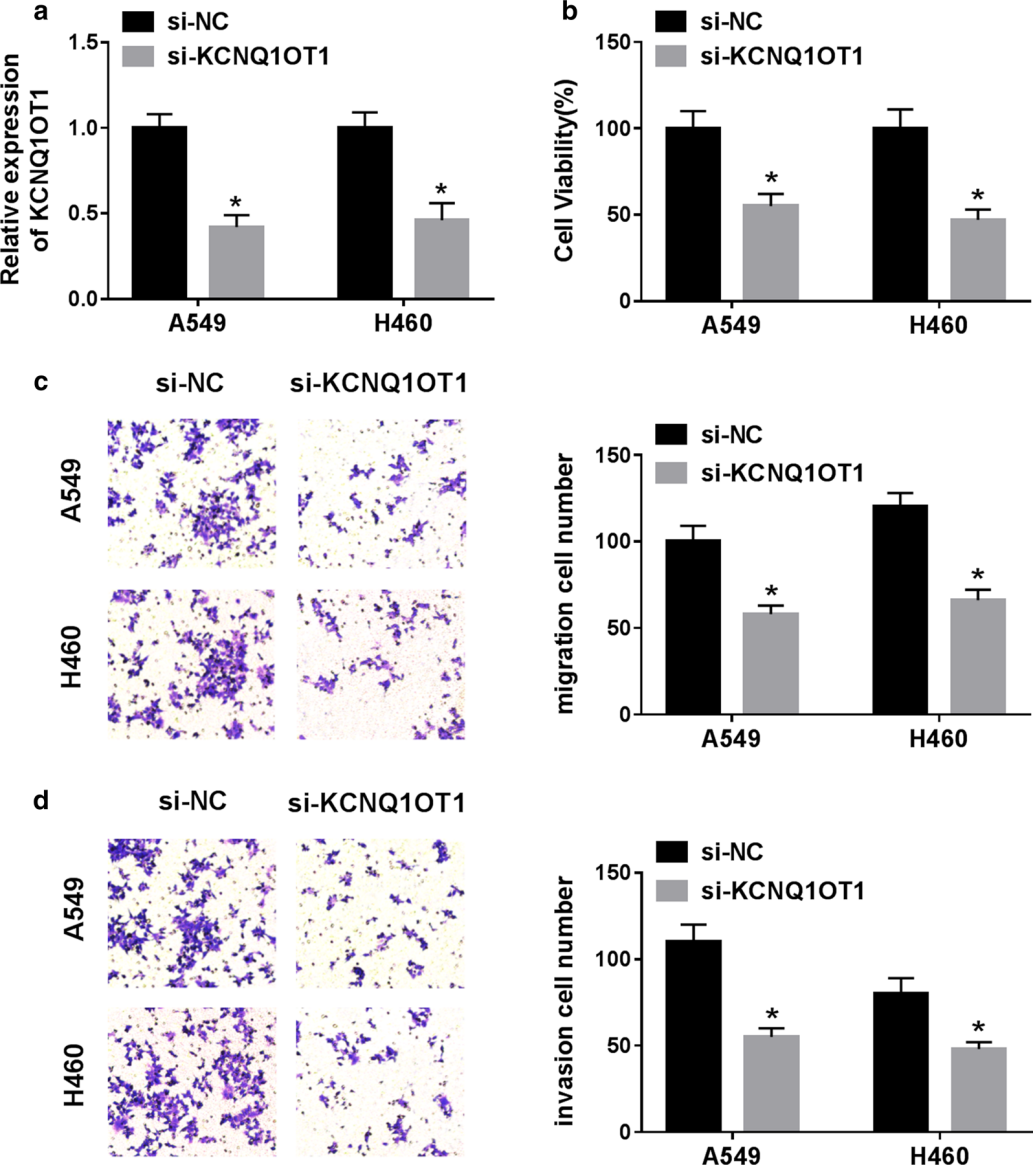
KCNQ1OT1 knockdown inhibited proliferation, migration and invasion of NSCLC cells. A549 and H460 cells were transfected with si-KCNQ1OT1 or the control si-NC. **a** The expression of KCNQ1OT1 was detected by qRT-PCR in transfected cells. **b** Cell proliferation was evaluated using CCT-8 assay. **c**, **d** The migrated and invaded cells were measured by transwell assay. *P < 0.05

To investigate the effects of KCNQ1OT1 on NSCLC progression, A549 and H460 cells were transfected with si-KCNQ1OT1, si-KCNQ1OT1#2, si-KCNQ1OT1#3 or si-NC. First, qRT-PCR results showed that the si-KCNQ1OT1 group had the most significant down-regulation after transfection with si-KCNQ1OT1, si-KCNQ1OT1#2 or si-KCNQ1OT1#3, so si-KCNQ1OT1 was selected for subsequent research (Fig. 2a and Additional file 1: Figure S1). CCK-8 assay and transwell assay exhibited that KCNQ1OT1 knockdown dramatically suppressed cell viability (Fig. 2b), migration (Fig. 2c) and invasion (Fig. 2d) in A549 and H460 cells. These data demonstrated that KCNQ1OT1 knockdown blocked cell proliferation, migration and invasion of NSCLC cells.

### KCNQ1OT1 directly targeted miR-129-5p in NSCLC cells

**Fig. 3:**
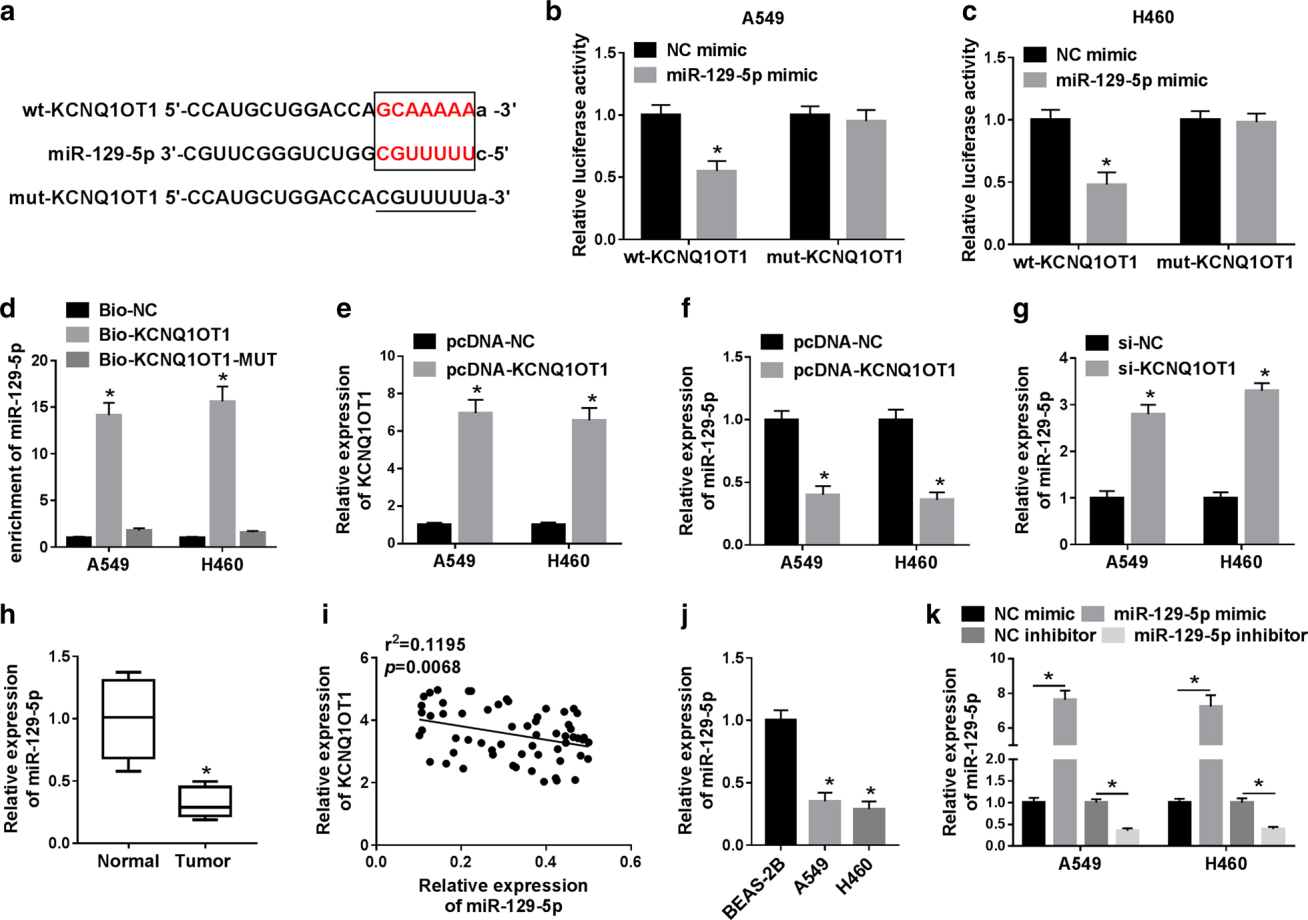
KCNQ1OT1 directly targeted miR-129-5p in NSCLC cells. **a** The putative binding sites of KCNQ1OT1 and miR-129-5p were shown. **b**, **c** Luciferase activity was examined in A549 and H460 cells co-transfected with wt-KCNQ1OT1 or mut-KCNQ1OT1 and miR-129-5p mimic or NC mimic. **d** RNA pull-down assay was performed to confirm the relationship between KCNQ1OT1 and miR-129-5p. **e** Transfection efficiency was measured using qRT-PCR in A549 and H460 cells introduced with pcDNA-NC or pcDNA-KCNQ1OT1. **f**, **g** A549 and H460 cells were transfected with pcDNA-NC, pcDNA-KCNQ1OT1, si-NC or si-KCNQ1OT1, and miR-129-5p expression was detected by qRT-PCR after transfection. **h** MiR-129-5p expression in normal tissues and NSCLC tissues was examined by qRT-PCR. **i** The correlation between KCNQ1OT1 and miR-129-5p was exhibited. **j** MiR-129-5p expression in BEAS-2B cells and NSCLC cell lines (A549 and H460) was detected by qRT-PCR. **k** MiR-129-5p level was examined by qRT-PCR in A549 and H460 cells transfected with NC mimic, miR-129-5 mimic, NC inhibitor or miR-129-5 inhibitor. *P < 0.05

To verify whether KCNQ1OT1 could act as a ceRNA by competitively binding miRNAs in NSCLC, we predicted that KCNQ1OT1 had putative binding sites with miR-129-5p by LncBase Predicted v.2 (Fig. 3a). For further validation, dual-luciferase reporter assay was performed. The results showed that cells co-transfected with wt-KCNQ1OT1 and miR-129-5p mimic had strikingly lower luciferase activity than other co-transfected complexes (Fig. 3b, c). Moreover, RNA pull-down assay further confirmed that KCNQ1OT1 bound to miR-129-5p (Fig. 3d). Besides, the overexpression efficiency of KCNQ1OT1 was determined by qRT-RCR (Fig. 3e and Additional file 2: Figure S2). Furthermore, KCNQ1OT1 overexpression significantly reduced miR-129-5p expression, and KCNQ1OT1 knockdown strikingly increased miR-129-5p expression in A549 and H460 cells (Fig. 3f, g). In addition, miR-129-5p expression was remarkably down-regulated in NSCLC tissues and cells (Fig. 3h, j), and was negatively correlated with KCNQ1OT1 expression in NSCLC tissues (Fig. 3i). Also, the overexpression efficiency and suppression efficiency of miR-129-5p were determined by qRT-PCR (Fig. 3k). These results revealed that KCNQ1OT1 directly bound to miR-129-5p in NSCLC.

### Inhibition of miR-129-5p reversed the effects of KCNQ1OT1 knockdown on proliferation, migration, invasion of NSCLC cells

**Fig. 4:**
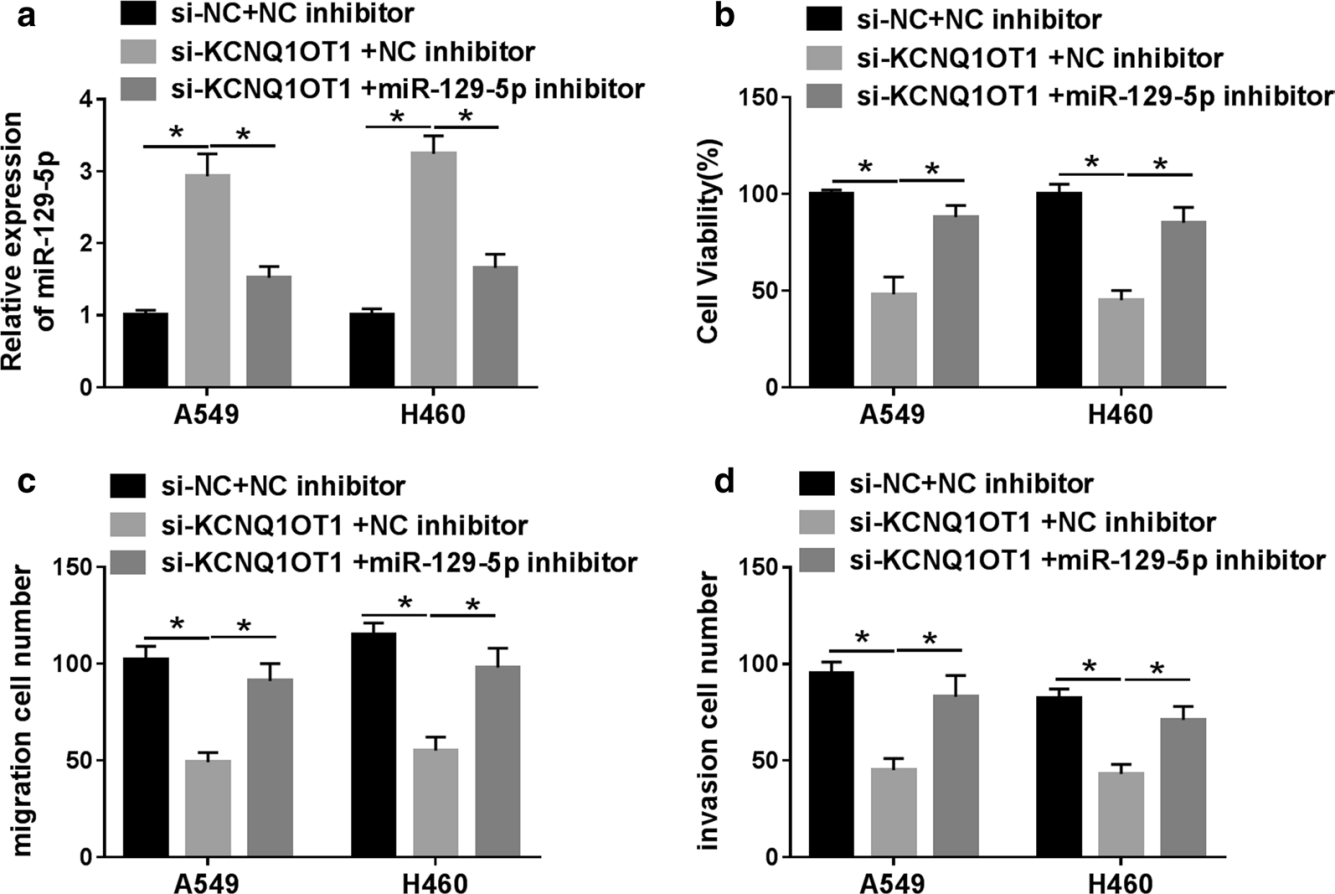
Inhibition of miR-129-5p reversed the effects of KCNQ1OT1 knockdown on NSCLC progression. (A-D) A549 and H460 cells were transfected with si-NC + NC inhibitor, si-KCNQ1OT1 + NC inhibitor or si-KCNQ1OT1 + miR-129-5p inhibitor. **a** The expression of miR-129-5p was detected by qRT-PCR. **b** Cell proliferation was detected by CCK-8 assay. **c** The migrated cells were measured by transwell assay. **d** The invaded capacity was evaluated by transwell assay. *P < 0.05

To further investigate the effects of miR-129-5p on NSCLC development, A549 and H460 cells were transfected with si-NC + NC inhibitor, si-KCNQ1OT1 + NC inhibitor or si-KCNQ1OT1 + miR-129-5p inhibitor. The results showed that transfection with miR-129-5p inhibitor attenuated the increase in miR-129-5p expression caused by KCNQ1OT1 silencing (Fig. 4a). Moreover, simultaneous knockdown of KCNQ1OT1 and miR-129-5p reversed the inhibitory effects of KCNQ1OT1 depletion on cell proliferation (Fig. 4b), migration (Fig. 4c) and invasion (Fig. 4d) of A549 and H460 cells. These results indicated that inhibition of miR-129-5p could abrogate the inhibitory effects of KCNQ1OT1 knockdown on NSCLC progression.

### MiR-129-5p targeted JAG1 in NSCLC cells

**Fig. 5:**
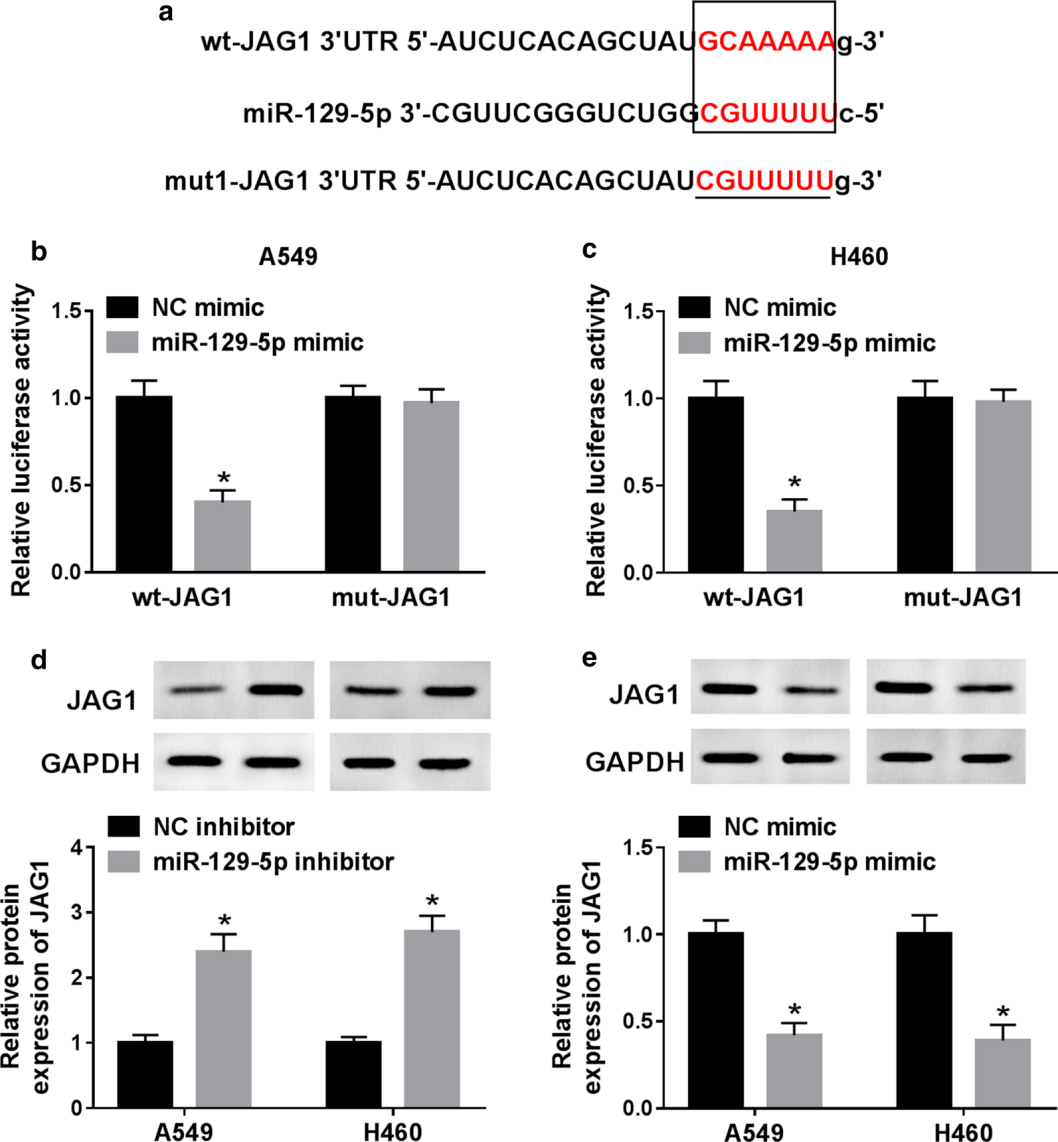
MiR-129-5p targeted JAG1 in NSCLC cells. **a** The putative binding sites of miR-129-5p and JAG1. **b**, **c** Luciferase activity was examined in A549 and H460 cells co-transfected with wt-JAG1 or mut-JAG1 and miR-129-5p mimic or NC mimic. **d** The protein level of JAG1 in A549 and H460 cells transfected with NC inhibitor or miR-129-5p inhibitor. **e** The protein level of JAG1 in A549 and H460 cells transfected with NC mimic or miR-129-5p mimic. *P < 0.05

StarBase v2.0 predicted that miR-129-5p might bind to JAG1 (Fig. 5a). Then, dual-luciferase reporter assay was performed to verify whether JAG1 was a target for miR-129-5p. The results suggested that miR-129-5p mimic significantly decreased the luciferase activity in A549 and H460 cells transfected with wt-JAG1, whereas luciferase activity could not be regulated when the binding sites were mutated (Fig. 5b, c). To further explore whether JAG1 was regulated by miR-129-5p, western blot results revealed that miR-129-5p inhibition obviously increased the protein level of JAG1, while miR-129-5p mimic markedly reduced the protein level of JAG1 in A549 and H460 cells compared to the negative control (Fig. 5d, e). These data suggested that JAG1 was a target gene of miR-129-5p.

### MiR-129-5p modulated NSCLC progression by targeting JAG1

**Fig. 6:**
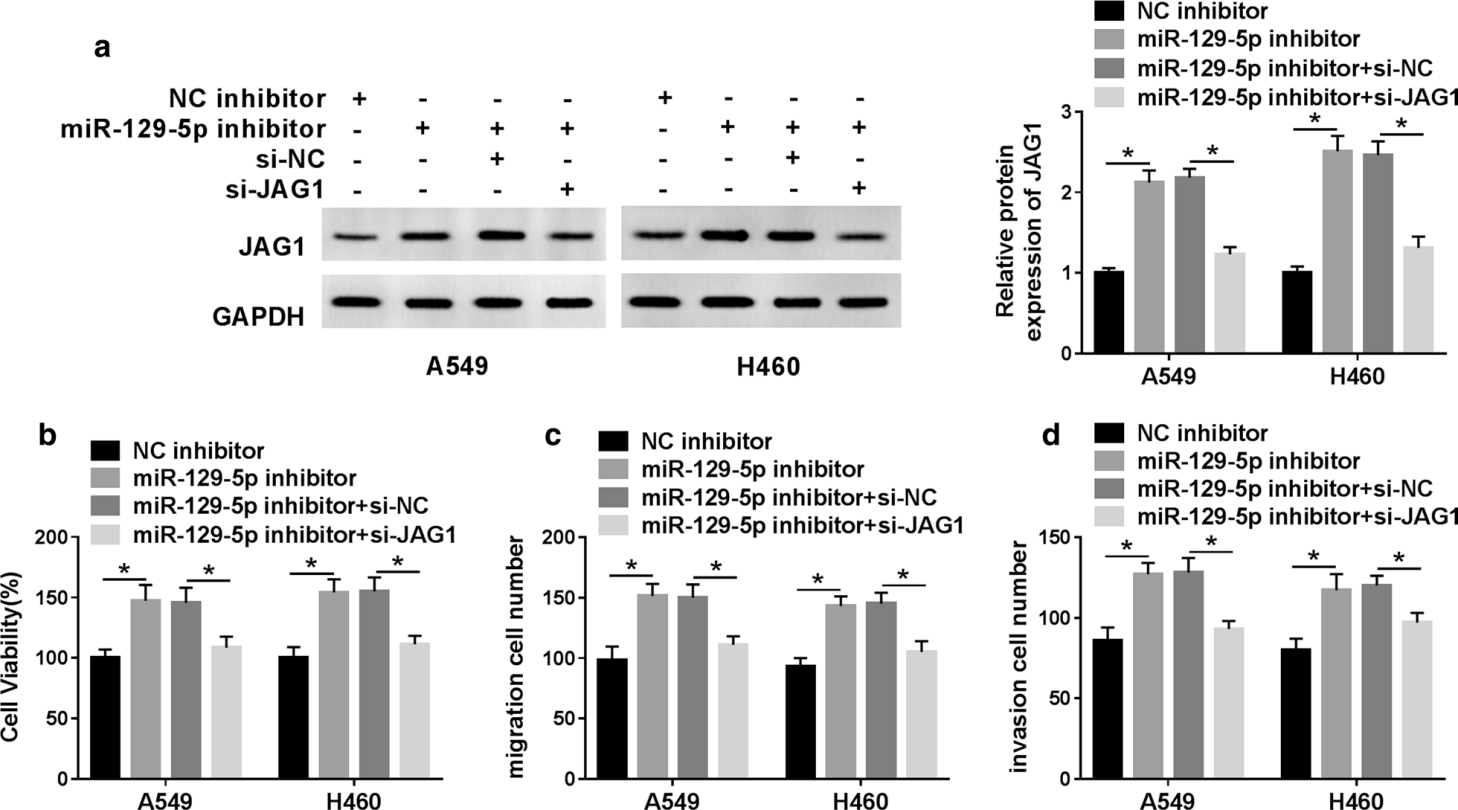
MiR-129-5p modulated NSCLC progression by targeting JAG1. (A-D) A549 and H460 cells were transfected with NC inhibitor, miR-129-5p inhibitor, miR-129-5p inhibitor + si-NC or miR-129-5p inhibitor + si-JAG1. **a** The protein level of JAG1 was detected by western blot. **b** Cell proliferation was detected by CCK-8 assay. **c** The migrated cells were measured by transwell assay. **d** Cell invasion was evaluated by transwell assay after transfection. *P < 0.05

To further determine the effects of miR-129-5p on NSCLC progression, A549 and H460 cells were transfected with NC inhibitor, miR-129-5p inhibitor, miR-129-5p inhibitor + si-NC or miR-129-5p inhibitor + si-JAG1. Firstly, western blot analysis revealed that transfection with si-JAG1 alleviated the elevation in JAG1 protein expression caused by miR-129-5p knockdown (Fig. 6a). Moreover, CCK-8 assay showed that down-regulation of miR-129-5p obviously increased cell viability, while the effect was eliminated by silencing of JAG1 (Fig. 6b). Transwell assay revealed that knockdown of miR-129-5p remarkably promoted cell migration and invasion, whereas the impact was reversed after transfection with si-JAG1 (Fig. 6c, d). These results indicated that miR-129-5p might suppress cell proliferation, migration and invasion in NSCLC by modulating JAG1.

### KCNQ1OT1 regulated JAG1 by sponging miR-129-5p in NSCLC cells

**Fig. 7:**
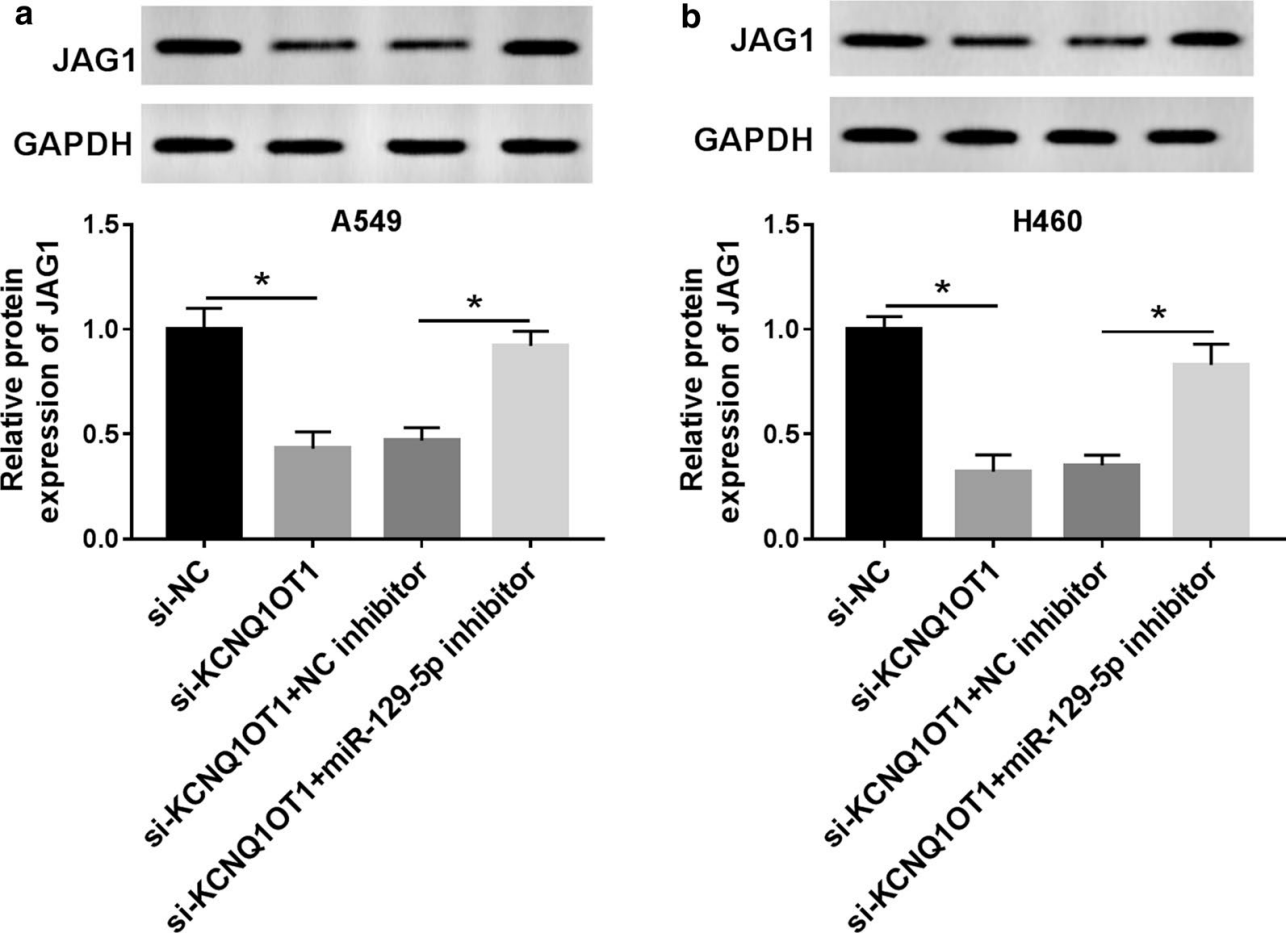
KCNQ1OT1 regulated JAG1 by sponging miR-129-5p in NSCLC cells. **a**, **b** A549 and H460 cells were transfected with si-NC, si-KCNQ1OT1, si-KCNQ1OT1 + NC inhibitor, or si-KCNQ1OT1 + miR-129-5p inhibitor. The protein level of JAG1 was detected by western blot. *P < 0.05

The western blot results revealed that KCNQ1OT1 knockdown significantly reduced the protein levels of JAG1, whereas the miR-129-5p inhibitor restored the JAG1 expression in A549 and H460 cells (Fig. 7a, b). Moreover, JAG1 expression was overtly increased in NSCLC tissues and was positively correlated with KCNQ1OT1 expression (Additional file 3: Figure S3A and AB). These results suggested that KCNQ1OT1 modulated JAG1 expression by regulating miR-129-5p in NSCLC cells.

## Discussion

With the great achievements in diagnosis and treatment, the survival time of NSCLC has increased, but the prognosis of NSCLC is still poor [18]. In recent years, the studies suggested that lncRNAs dysregulation was associated with the progression of cancers, including NSCLC [19]. To elucidate the molecular mechanism of lncRNAs in the prognosis of NSCLC is meaningful.

Recent studies exhibited that lncRNA KCNQ1OT1 was upregulated in various cancers. A previous study suggested that KCNQ1OT1 facilitated tumor growth by competitively sponging miR‑504 and up-regulating cyclin‑dependent kinase 16 (CDK16) in hepatocellular carcinoma [20]. A previous research revealed that KCNQ1OT1 was upregulated in early stage lung cancer and was associated with prognosis in LC patients by suppressing cell proliferation [21]. Furthermore, Dong et al. demonstrated that KCNQ1OT1 was markedly upregulated in NSCLC tissues and cells, and promoted NSCLC cells proliferation, migration, and invasion by regulating the KCNQ1OT1/miR-27b-3p/HSP90AA1 axis [22]. Consistent with previous study, the level of KCNQ1OT1 in NSCLC tissues and cell lines was dramatically higher than that in non-tumor tissues and cells. These results revealed that KCNQ1OT1 might exhibit vital roles in NSCLC development and progression.

It has been reported that lncRNAs could be used as competitive endogenous RNAs (ceRNAs) [23]. This study showed that KCNQ1OT1 could competitively bind to miR-129-5p. MiR-129-5p has been reported as an anti-tumor role in many cancers. In ovarian cancer, miR-129-5p was significantly downregulated in OC tissues and cells, and miR-129-5p acting as tumor suppressor inhibited cell proliferation and promoted apoptosis of OC cell by attenuating the effects of PCAT-1 [24]. In osteosarcomas, MALAT1 increased stem cell-like properties by regulating the expression of RET via sponging miR-129-5p [25]. Moreover, the effects of miR-129-5p in NSCLC have also been reported. MiR-129-5p suppressed NSCLC stemness and chemoresistance by targeting DLK1 [26]. In our study, we demonstrated that miR-129-5p was dramatically down-regulated in NSCLC tissues and cells. Meanwhile, miR-129-5p had binding sites with KCNQ1OT1. Inhibition of miR-129-5p could abolish the effects of KCNQ1OT1 knockdown on the progression of NSCLC.

JAG1 is a Notch ligand that plays a vital role in a variety of signaling pathways [27]. Recent evidence suggests that JAG1 was an oncogene in NSCLC by inducing cell metastasis [28]. In this study, we found that JAG1 was inhibited by miR-129-5p in NSCLC, and JAG1 knockdown reversed the effects of miR-129-5p inhibition. In short, KCNQ1OT1 regulated JAG1 expression by sponging miR-129-5p in NSCLC cells.

## Conclusion

In conclusion, this study suggested that KCNQ1OT1 and JAG1 were upregulated, while miR-129-5p was down-regulated in NSCLC tissues and cells. Knockdown of KCNQ1OT1 significantly inhibited proliferation, migration and invasion of NSCLC cells. Meanwhile, miR-129-5p inhibition reversed the effects of KCNQ1OT1 knockdown on the progression of NSCLC. In a word, we found that KCNQ1OT1 promoted the NSCLC progression by regulating the KCNQ1OT1/miR-129-5p/JAG1 axis, which provides therapeutic targets for NSCLC.

## Supplementary information

**Additional file 1: Figure S1.** The knockdown efficiency of KCNQ1OT1 was determined by qRT-PCR in A549 and H460 cells transfected with si-KCNQ1OT1, si-KCNQ1OT1#2 or si-KCNQ1OT1#3. *P < 0.05.

**Additional file 2: Figure S2.** The expression of KCNQ1OT1 was detected in BEAS-2B cells, NSCLC cells and NSCLC cells transfected with pcDNA-NC or pcDNA-KCNQ1OT1. *P < 0.05.

**Additional file 3: Figure S3.** (A) The expression of JAG1 mRNA in normal tissues and NSCLC tissues was detected by qRT-PCR. (B) The correlation between JAG1 mRNA and KCNQ1OT1 in NSCLC tissues was analyzed by Spearman’s correlation analysis. *P < 0.05.

## Data Availability

All data generated or analyzed during this study are included in this published article.

## Abbreviations

NSCLC: Non-small cell lung cancer
SCLC: Small cell lung cancer
CCA: Cholangiocarcinoma
miRNAs: MicroRNAs
