# Supplementary figures and images for: LncRNA KCNQ1OT1 promotes cell proliferation, migration and invasion via regulating miR-129-5p/JAG1 axis in non-small cell lung cancer
Source: Cancer Cell Int. 2020 May 1;20:144. doi: 10.1186/s12935-020-01225-8 (PMC7195752; doi:10.1186/s12935-020-01225-8)

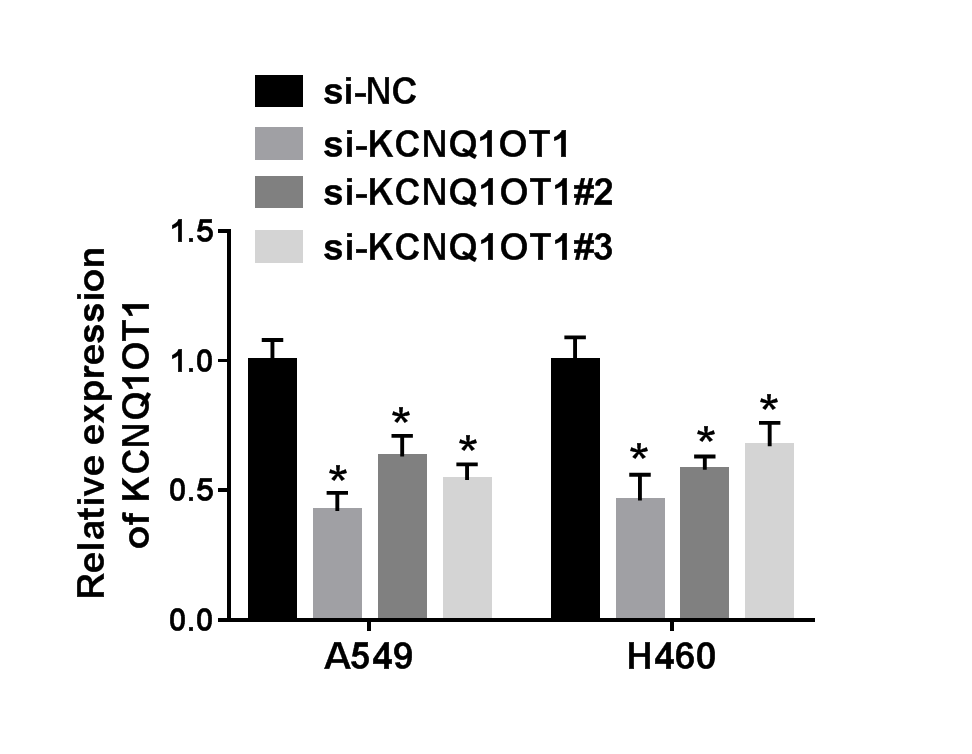

Supplement: Supplementary file 1 — Additional file 1: Figure S1. The knockdown efficiency of KCNQ1OT1 was determined by qRT-PCR in A549 and H460 cells transfected with si-KCNQ1OT1, si-KCNQ1OT1#2 or si-KCNQ1OT1#3. *P < 0.05. [file 12935_2020_1225_MOESM1_ESM.tif]

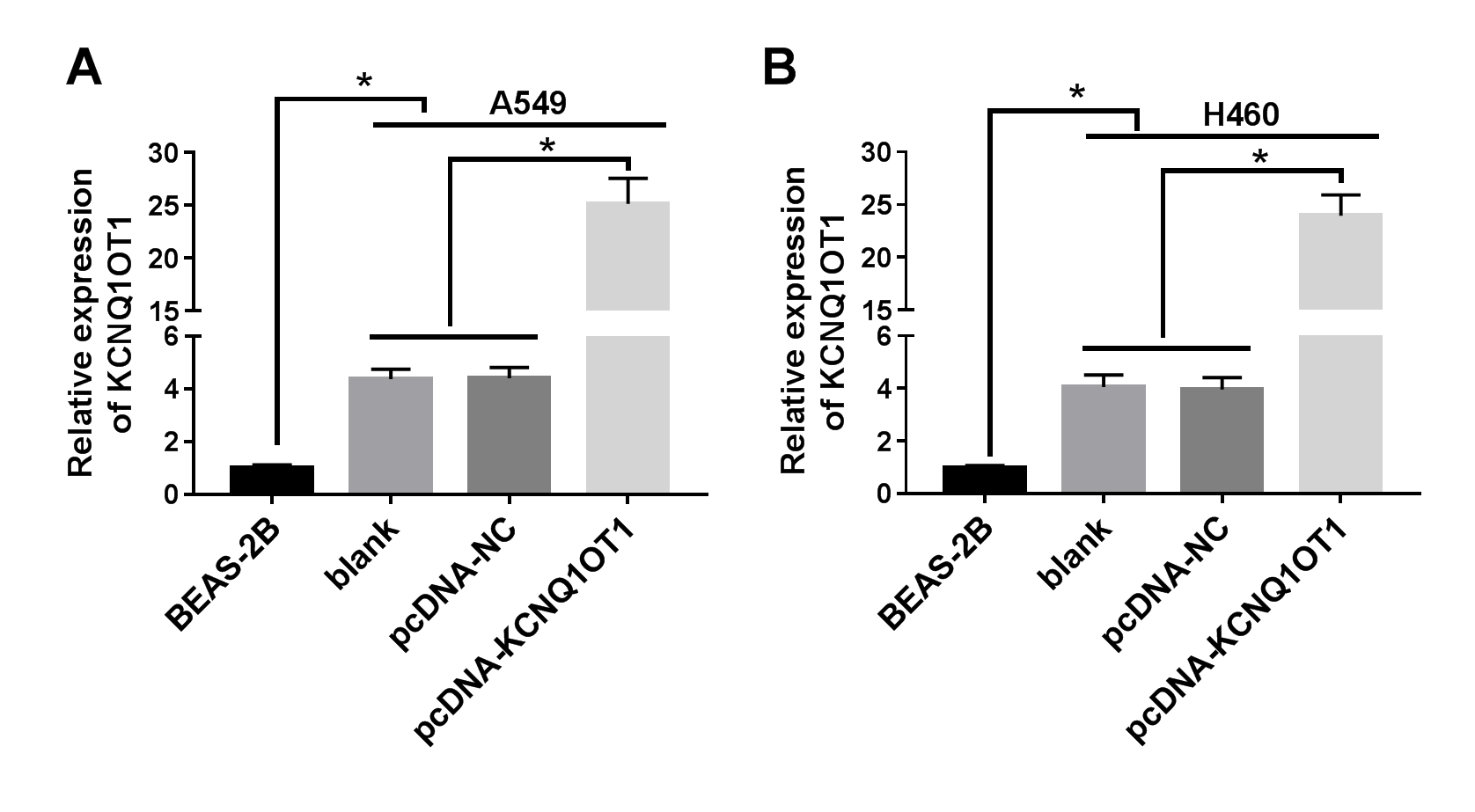

Supplement: Supplementary file 2 — Additional file 2: Figure S2. The expression of KCNQ1OT1 was detected in BEAS-2B cells, NSCLC cells and NSCLC cells transfected with pcDNA-NC or pcDNA-KCNQ1OT1. *P < 0.05. [file 12935_2020_1225_MOESM2_ESM.tif]

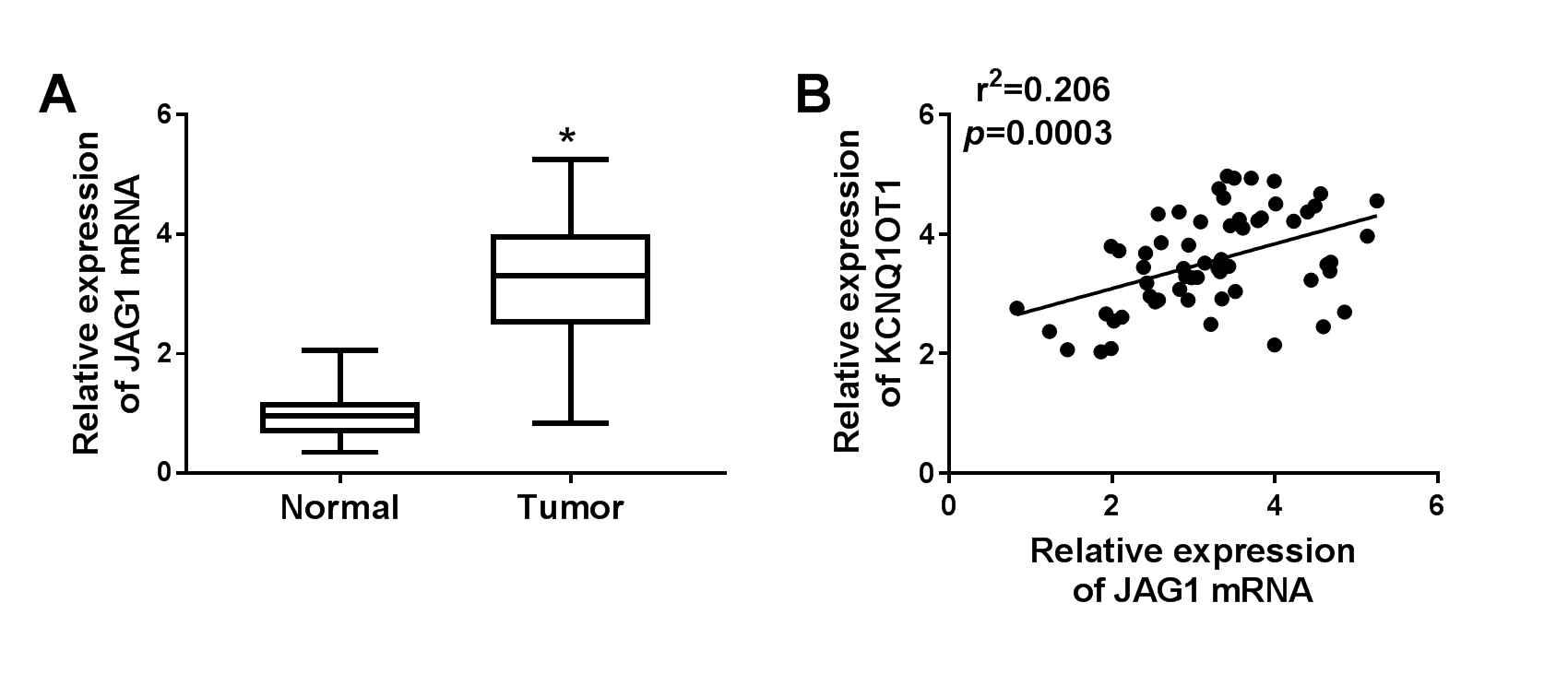

Supplement: Supplementary file 3 — Additional file 3: Figure S3. (A) The expression of JAG1 mRNA in normal tissues and NSCLC tissues was detected by qRT-PCR. (B) The correlation between JAG1 mRNA and KCNQ1OT1 in NSCLC tissues was analyzed by Spearman’s correlation analysis. *P < 0.05. [file 12935_2020_1225_MOESM3_ESM.tif]
